# MI-63: A novel small-molecule inhibitor targets MDM2 and induces apoptosis in embryonal and alveolar rhabdomyosarcoma cells with wild-type p53

**DOI:** 10.1038/sj.bjc.6605199

**Published:** 2009-08-25

**Authors:** J A Canner, M Sobo, S Ball, B Hutzen, S DeAngelis, W Willis, A W Studebaker, K Ding, S Wang, D Yang, J Lin

**Affiliations:** 1 Department of Pediatrics, Nationwide Children's Hospital, Columbus, OH, USA; 2 Center for Childhood Cancer, Research Institute at Nationwide Children's Hospital, Columbus, OH, USA; 3 Molecular, Cellular, and Developmental Biology Program, Columbus, OH, USA; 4 Molecular Therapeutics Program, University of Michigan Comprehensive Cancer Center, Ann Arbor, MI, USA; 5 Ascenta Therapeutics, Inc., San Diego, CA, USA

**Keywords:** small molecule inhibitors, MDM2, p53, rhabdomyosarcoma, apoptosis

## Abstract

**Background:**

Interruption of the role of p53s as a tumour suppressor by MDM2 may be one of the mechanisms by which cancer cells evade current therapy. Blocking the inhibition of wild-type p53 by MDM2 in cancer cells should reactivate p53's tumour suppressor functions and enhance current cancer treatments. MI-63 is a novel non-peptide small molecule that has shown strong binding affinity (K_*i*_=3 nM) for MDM2; however, its effects on paediatric cancer cells and the specific mechanism of tumour suppressor reactivation have not been evaluated.

**Methods:**

Rhabdomyosarcoma (RMS), the most common childhood soft tissue sarcoma, expresses either wild-type or mutant p53 protein. We examined the inhibitory effects of MI-63 in embryonal RMS (ERMS) and alveolar RMS (ARMS) cell lines expressing wild-type or mutated p53.

**Results:**

Treatment with MI-63 reduced cell viability by 13.4% and by <1%, respectively, at 72 h in both RH36 and RH18 cell lines expressing wild-type p53. In contrast, RH30 and RD2 cells expressing p53 mutants are resistant to MI-63 treatment. An increased expression of p53, p21^WAF1^, and Bax protein was observed after treatment with MI-63 in RMS cells with wild-type p53, and apoptosis was confirmed by cleaved PARP and caspase-3 expression. However, RD2 and RH30 RMS cells, as well as human normal skeletal muscle cells, showed a minimal increase in p53 signalling and no induction of cleaved PARP and caspase-3. MI-63 was compared with Nutlin-3, a known MDM2 inhibitor, and was found to be more potent in the inhibition of cell proliferation/viability. Further, synergy was observed when MI-63 was used in combination with doxorubicin.

**Conclusion:**

These results indicate that MI-63 is a potent therapeutic agent for RMS cells expressing wild-type p53 protein.

Rhabdomyosarcoma (RMS) is the most common paediatric soft tissue sarcoma with a yearly incidence of approximately 4.6 cases per million (Ries and SEER Program (National Cancer Institute (US)), 1999; Breneman *et al*, 2003). Although a highly heterogeneous tumour, the most common histologic subtypes of RMS are embryonal rhabdomyosarcoma (ERMS) and alveolar rhabdomyosarcoma (ARMS) (Davicioni *et al*, 2006). IRS-III outcome data indicate that, with multimodal therapy, cure rates have improved to nearly 70%; however, survival of children with aggressive subtypes (i.e., ARMS) or with metastatic disease continues to be poor (Crist *et al*, 1995; Pappo *et al*, 1995; Taylor *et al*, 2000; Breneman *et al*, 2003). Attempts to improve survival have not been successful with different combinations of known chemotherapeutic agents; thus, the need for novel therapies (Raney *et al*, 2001).

The p53 protein regulates cell cycle, repairs DNA, and initiates apoptosis (Ding *et al*, 2006). Thus, p53's role as a tumour suppressor is well known and studied. p53 is mutated in paediatric malignancies in approximately 50% of cases; however, the frequency of mutations differs among tumour types (Taylor *et al*, 2000; Ding *et al*, 2006). The remaining tumours that retain wild-type p53 develop alternative mechanisms that disrupt normal function and lead to malignant proliferation (Tovar *et al*, 2006). Overexpression of MDM2, a regulator of p53 transcription, leads to abnormal protein–protein interactions and inhibits p53 activity.

Stability of the p53–MDM2 interaction is necessary for cell-cycle arrest and apoptosis. When the balance between MDM2 and p53 is disrupted, MDM2 upregulation inhibits the function of p53 as a tumour suppressor (Chen *et al*, 1995; Taylor *et al*, 2000; Takahashi *et al*, 2004; Tovar *et al*, 2006). Specifically, reports identify approximately one-third of sarcomas with MDM2 overexpression and thus, an interruption of p53 activity (Keleti *et al*, 1996; Taylor *et al*, 2000). RMS tumour cells with both mutated and wild-type p53 have been identified (Keleti *et al*, 1996; Taylor *et al*, 2000). Although the frequency of wild-type p53 is unclear, RMS tumours with wild-type p53, with or without MDM2 amplification, are a good target for small-molecule inhibitors, potentially allowing for reactivation of protein signalling.

MI-63, a novel, non-peptide small-molecule inhibitor, shows promise as an inhibitor of the p53–MDM2 interaction in prostate cancer cells with wild-type p53; however, it has not been tested in paediatric sarcomas (Ding *et al*, 2006). Previously, non-peptide small-molecule inhibitors of the p53–MDM2 interaction have targeted three, well-described, hydrophobic residues (Phe19, Trp23, and Leu26); however, the MI63 design includes a fourth residue (Leu22) that has an important role in p53 binding to MDM2 (Lin *et al*, 1994; Kussie *et al*, 1996; Ding *et al*, 2006). Incorporating this residue, MI-63 now has a greater binding affinity to MDM2, K_*i*_ value of 3 nM, and high selectivity in blocking p53–MDM2 interaction when compared with previously described non-peptide inhibitors (Ding *et al*, 2006). MI-63 has been shown to reactivate wild-type p53 function and decrease cancer cell proliferation (Ding *et al*, 2006).

To investigate the effect of MI-63 on paediatric sarcomas, specifically RMS, and the reactivation of p53 cell signalling, we designed an *in vitro* study examining cancer cell proliferation and presence of apoptosis after exposure to the inhibitor. ERMS and ARMS cell lines with wild-type p53 were treated with MI-63, and cell proliferation and levels of p53, p21^WAF1^, Bax, cleaved PARP, and cleaved caspase-3 protein were evaluated. On the basis of MI-63 binding potential and data presented in adult tumours, we expected a reactivation of the p53 signalling pathway and subsequent RMS cell death.

In addition, our experiment evaluated the synergism between the known cytotoxic agent, doxorubicin, and MI-63. Although many chemotherapeutic agents have been used in an attempt to treat RMS, toxicities and treatment failures provide motivation for the discovery of new agents and combinations. Combined chemotherapy allows the treatment of cancer cells through different mechanisms, with goals of improving outcome and minimising toxicity. Evidence of enhanced tumour suppression would be encouraging, and may indicate an ability to achieve similar or improved treatment outcomes with decreased toxicities.

## Materials and methods

### Human cancer cell lines

HCT-116 p53 +/+ and HCT-116 p53 −/− colon cancer cells were kindly provided by Berg Volgelsten's laboratory at the John Hopkins University. Both colon cancer cell lines were cultured in DMEM media with L-glutamine (Mediatech, Inc.; Herndon, VA, USA) containing 10% FBS (Gibco; Grand Island, NY, USA) and 1% by volume penicillin/streptomycin (Invitrogen Life Technologies; Carlsbad, CA, USA). ERMS cell, RH36, and ARMS cells, RH18 and RH30, were a gift from Peter Houghton at St. Judes Children's Research Hospital. The RD2 tumour cell line (ERMS) was a gift from Brett Hall at The Ohio State University. All RMS cell lines were cultured in RPMI-1640 media with L-glutamine (Mediatech, Inc.) containing 10% FBS and 1% by volume penicillin/streptomycin (Invitrogen Life Technologies). Human skeletal muscle cells (HMSS) were purchased from Lonza Walkersville Inc (Walkersville, MD, USA). Normal human cells were cultured in SkBM-2 medium containing hEGF, Dexamethasone, L-glutamine, FBS and Gentamicin/Amphotericin-B in pre-mixed aliquots as per manufacturer's instructions. All cell lines were cultured until confluent and maintained in humidified incubators at 37°C and 5% CO_2_.

### Small-molecule compound

**Figure 1:**
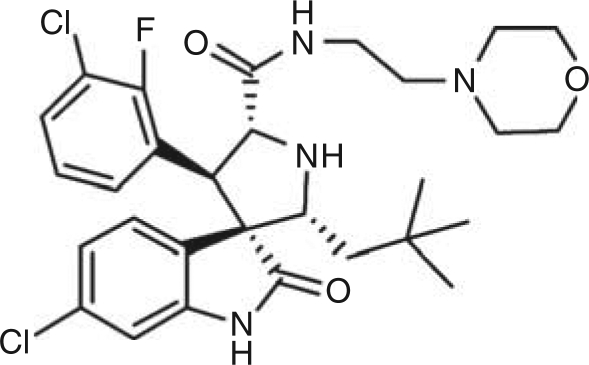
Chemical structure of MI-63.

The small-molecule inhibitor, MI-63, was provided by Dr Shaomeng Wang's laboratory at the University of Michigan (Figure 1). MI-63 was dissolved in DMSO (ATCC; Manassas, VA, USA) to a 10 mM stock solution and stored at −20°C.

### MTT cell viability assay

Cell lines were plated at 7000 cells/well (100 *μ*l/well) into a 96-well plate and incubated overnight. The medium was removed and replaced with fresh DMEM or RPMI either by itself, with DMSO (7 *μ*l dissolved in 10 ml of DMEM or RPMI), or with MI-63 concentrations of 5 *μ*M or 10 *μ*M. Treated cells were incubated up to 72 h. Each well was then incubated with Thiazolyl Blue Tetrazolium Bromide (MTT; Sigma; St Louis, MO, USA) for 3 h. Formazan crystals were solubilised with a detergent containing 1 : 1 dilution of *N*,*N*-demethylformamide (Sigma) and 20% SDS, wt/vol (Fisher Scientific; Fair Lawn, NJ). Spectrophotometric absorbance of the plate was recorded at 595 nm using an EL808 Ultra Microplate Reader (Bio-Tek Instruments, Inc; Winooski, VT, USA). Data were collected using KC Junior software (Bio-Tek Instruments, Inc) and exported to Microsoft Excel for analysis. Half-Maximal inhibitory concentrations (IC_50_) were determined using Sigma Plot 9.0 software (Systat Software Inc., San Jose, CA, USA) with the 4-parameter logistic function standard curve analysis for dose response.

### Western blot analysis

Cell lines were plated in 10 cm plates at a concentration of 400 000 cells/plate and were allowed to adhere overnight. Cells were incubated in DMEM, RPMI, or SkBM-2 medium (HMSS), with growth factors as above, for 24 h (RH18 cells were incubated for 48 h). After treating with 5 and 10 *μ*M of MI-63, the cells were washed with PBS and lysed in SDS lysis buffer (62.5 mM Tris-HCL, 2% w/v SDS, 10% glycerol, 50 nM DTT, 0.02% w/v bromphenol blue). Whole-cell lysates were boiled at 95°C in water for 5 min and separated on 10% or 12% glycine-based SDS PAGE gels. After electrophoresis, samples were transferred to a polyvinylidine fluoride membrane (PVDF). The PVDF membrane was blocked in 5% milk in 1% Tween-20 in TBS (TBST) for an hour at room temperature. The membrane was then incubated overnight with a primary antibody at 4^°^C in 2.5% milk in TBST. Thereafter, blots were incubated for over an hour with a fluorescein-linked secondary antibody, and then for 30 min with an alkaline-phosphatase-linked anti-fluorescein tertiary antibody. Protein bands were visualised using an ECL chemiluminescent substrate and the Storm Scanner. Primary antibodies for p53, p21^WAF1^, MDM2, cleaved caspase-3, cleaved PARP, GAPDH, and Bax were obtained from Cell Signaling Technologies (Danvers, MA, USA). Secondary antibodies were obtained from Jackson Immuno Research Laboratories (West Grove, PA, USA) and the tertiary antibody was purchased from Sigma-Aldrich Inc. (St Louis, MO, USA).

### Determination of the half-life of p53

RH36 cells were treated with either DMSO or 5 *μ*M MI-63 and incubated for 24 h. The cells were then treated with 50 *μ*g/ml of cycloheximide (Sigma) and harvested at 0, 60, and 120 min as described above. Western blot analysis was carried out to determine the levels of p53.

### Determination of combinatorial effects

Doxorubicin and MI-63 synergy with regard to growth inhibition were determined as set forth by Chou and Talalay, (1984). Briefly, the log (*fa*/*fu*) was plotted against the concentration (D) for each compound, alone or in combination, where *fa* is the fraction affected and *fu* is the fraction unaffected (1-*fa*) of cells at each concentration. Calcusyn software (Biosoft, Ferguson, MO, USA), programmed in accordance with the methods described by Chou (Chou and Talalay, 1984), was used to determine the combinational index (CI) for each concentration of drug mixture used. A value CI less than 1 represents synergism between doxorubicin and MI-63. A value equal to/greater than 1 represents additive effects and antagonistic effects, respectively.

## Results

### MI-63 induces p53 and downstream cell signalling in HCT-116 p53+/+

**Figure 2:**
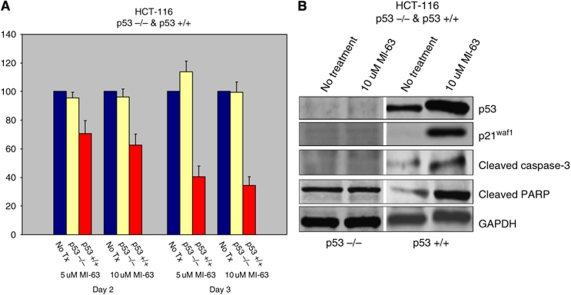
MI-63 treatments of HCT-116 p53 +/+ and HCT-116 p53 −/− cells. (**A**) MTT analysis indicates decreased cell proliferation with MI-63 in cells with wild-type p53. (**B**) A measure of 50 *μ*g of total protein from HCT-116 cell lysates was subjected to SDS polyacrylamide gel electrophoresis (PAGE) and transferred to PVDF membrane. Membranes were blotted with p53, p21^WAF1^, cleaved caspase-3, and GAPDH antibodies.

The colon cancer cell lines, HCT-116 p53+/+ and p53−/−, were evaluated for p53 specificity. HCT-116 p53+/+ cells incubated with 5 and 10 *μ*M of MI-63 (Figure 1) had decreased cell proliferation at days 2 (70.5%±9.21 and 62.7%±7.7, respectively) and 3 (40.4%±7.57 and 34.2%±6.33, respectively) using MTT assay (Figure 2A). Increases in p53, p21^WAF1^, cleaved caspase-3, and cleaved PARP proteins were found on western blot analysis. In contrast, HCT-116 p53−/− cells incubated with 5 and 10 *μ*M of MI-63 showed a minimal change in cellular proliferation on day 2 (95.1%±3.91 and 96.1%±5.56, respectively) and day 3 (113.6%±7.66 and 99.6%±7.07, respectively). Western blot analysis showed no evidence of p53, p21^WAF1^, or cleaved caspase-3 induction, and cleaved PARP was not increased. GAPDH demonstrated equal protein loading (Figure 2B).

### MI-63 reactivates p53 protein and induces apoptosis in RMS

**Figure 3:**
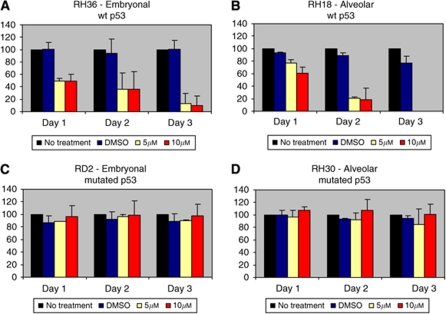
The effect of MI-63 on the cell viability of ERMS/ARMS cells. (**A**, **B**) Rhabdomyosarcoma cells with wild-type p53, RH36 and RH18, showing decreased cell proliferation when treated with MI-63. (**C**, **D**) Rhabdomyosarcoma cells with mutated p53, RD2 and RH30, showing decreased cell proliferation when treated with MI-63.

An ERMS cell line with wild-type p53, RH36, incubated with 5 and 10 *μ*M of MI-63 for 1–3 days, was evaluated. MTT assay showed decreased cell proliferation/viability across 3 days of exposure with 5 and 10 *μ*M of MI-63 (48.7%±5.41 and 48.7%±11.02 on day 1; 35.9%±26.56 and 35.6%±28.47 on day 2; 13.4%±16.11 and 9.8%±15.30 on day3) (Figure 3A). Similarly, a histologic ARMS cell line with wild-type p53, RH18, was incubated with 5 and 10 *μ*M of MI-63 for the same duration. MTT assay identified decreased cell viability in RH18 cells (77.3%,±5.44 and 60.9%±9.83 on day 1; 20.9%±2.05 and 18.2%±18.74 on day 2; <1%±0 and <1%±0 on day3) (Figure 3B).

ERMS and ARMS cell lines, RD2 and RH30, respectively, with mutated p53 were used as negative controls. MTT showed minimal changes in cell viability at both concentrations. RD2 cells treated for 1, 2, and 3 days continued to proliferate with 5 and 10 *μ*M of MI-63 treatments (88.5%±0.42 and 96.3%±18.03 on day 1; 96.3%±3.82 and 98.4%±23.48 on day 2; 90%±1.13 and 97.3%±18.38 on day3) (Figure 3C). Further, RH30 cells treated with MI-63 also showed similar results (97%±10.59 and 107.4%±5.20 on day 1; 91.8%±11.81 and 107.5%±17.64 on day 2; 84.3%±24.88 and 100.4%±17.18 on day3) (Figure 3D).

**Figure 4:**
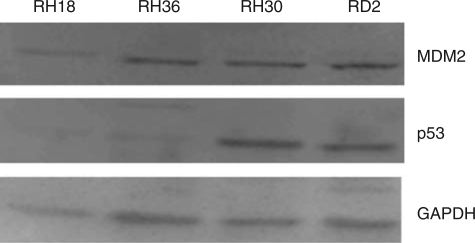
Baseline levels of MDM2 and p53 expression in wild-type p53, RH18 and RH36, and mutant p53, RH30 and RD2, rhabdomyosarcoma cells.

**Figure 5:**
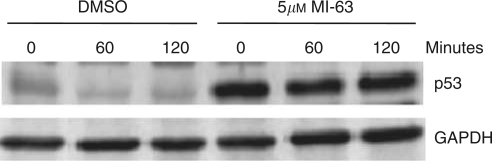
The effect of MI-63 on p53 half-life in RH36 cells. Blocking MDM2 activity inhibits p53 degradation resulting in an increased p53 half-life.

**Figure 6:**
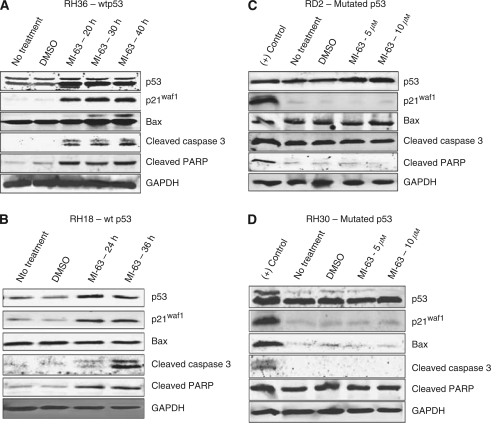
Western blot analysis of ERMS/ARMS cells treated with MI-63. (**A**, **B**) Rhabdomyosarcoma cells with wild-type p53, RH36 and RH18, indicate increased p53, downstream targets, p21^WAF1^ and Bax, as well as cleaved caspase-3 and cleaved PARP, at 20, 30, and 40 h after treatment with 10 *μ*M of MI-63. (**C**, **D**) Rhabdomyosarcoma cells with mutated p53, RD2 and RH30, indicate no increase of p53, downstream target p21^WAF1^, cleaved caspase-3, and cleaved PARP at 24 and 36 h after treatment with 10 *μ*M of MI-63.

The baseline levels of p53 and MDM2 were assessed in RH18 and RH36 cells with wild-type p53 and in RH30 and RD2 cells expressing mutant p53, which have higher levels of p53 (Figure 4). The half-life of p53 in wild-type cells is short (Cinelli *et al*, 1998). This is in part due to the role of MDM2 as an E3 ubiquitin ligase, mediating a proteasomal degradation of p53 (Honda *et al*, 1997; Fuchs *et al*, 1998). The degradation of the p53 protein by MDM2 is inhibited in RH36 cells after treatment with MI-63, resulting in an increased p53 half-life (Figure 5). Both wild-type p53-expressing cell lines were treated with 10 *μ*M of MI-63. Western blot analysis indicated an induction of p53. RH36 cells were treated for 20, 30, and 40 h. Although baseline levels of p53 were present in the non-treated cells, levels increased after treatment. Antibodies for p21^WAF1^ were strongly positive when compared with those with no treatment (Figure 6A). The apoptotic pathway was reactivated, displayed by the increased presence of Bax protein by 30 h after treatment, and by the presence of both cleaved caspase-3 and cleaved PARP. RH18 cells expressing wild-type p53 were treated for 24 and 36 h and the findings were comparable (Figure 6B). p53 proteins increased from baseline with exposure to MI-63. Induction of the p21^WAF1^ protein was evident. Baseline levels of Bax protein were unchanged; however, downstream apoptosis proteins, cleaved caspase-3, and cleaved PARP were increased by 36 h (Figure 6B). In contrast, western blot analyses of both RD2 and RH30 cells expressing mutated p53 treated with MI-63 showed no change from untreated cells when p53, p21^WAF1^, Bax, cleaved caspase-3, and cleaved PARP proteins were evaluated (Figures 6C and D). These results indicate that MI-63 selectively induces apoptosis in rhabdomysarcoma cells expressing wild-type p53 but not in rhabdomysarcoma cells expressing mutated p53.

### MI-63 does not induce apoptosis in normal human muscle cells (HSMM)

**Figure 7:**
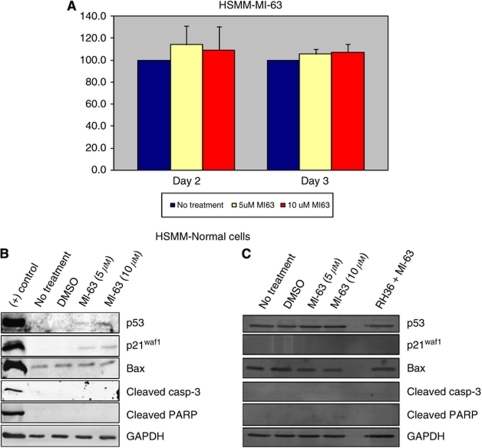
MI-63 treatments of HSMM cells. (**A**) MTT analysis shows minimal changes in cell proliferation when normal human skeletal muscle cells (HSMM) are treated with MI-63. (**B**) Western blot analysis of HSMM cells grown in SkBM-2 medium with growth factors. (**C**) Western blot analysis of HSMM cells grown in DMEM supplemented with 10% FBS. A measure of 50 *μ*g of total protein from HSMM cell lysates was subjected to SDS PAGE and transferred to PVDF membrane. Membranes were blotted with p53, p21^WAF1^, Bax, cleaved caspase-3, cleaved PARP, and GAPDH antibodies. The results show an increase in p53 protein and p21^WAF1^ at higher doses and prolonged exposure; however, there is no apparent reactivation of apoptosis.

Normal human skeletal muscle cells, HSMM, were incubated for 2 and 3 days with 5 and 10 *μ*M MI-63. Cell viability was maintained despite the treatment with 5 and 10 *μ*M MI-63 using MTT analysis (114%±17.1 and 109.1%±21.3 on day 2; 105.4%±4.8 and 107.2%±7.3 on day 3) (Figure 7A). Western blots showed a minimal presence of p53 and the downstream p21^WAF1^ protein after treatment with 10 *μ*M; however, there was no evidence of apoptosis, as no cleaved caspase-3 or cleaved PARP was identified in the SkBM-2 (Figure 7B) or DMEM (Figure 7C) medium.

### MI-63 is a more potent MDM2 inhibitor than Nutlin-3

**Figure 8:**
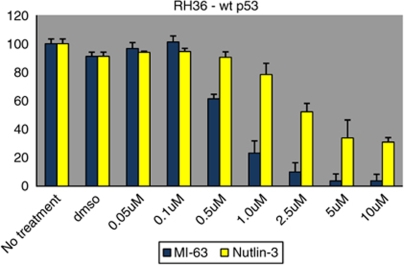
A comparison of the effects of MI-63 and Nutlin-3 on the viability of RH36 cells. Treatment with MI-63 results in a greater decrease in cell proliferation/viability.

MI-63 has been shown to have a greater binding affinity for MDM2 and to have a greater effect on cell viability than Nutlin-3 (Ding *et al*, 2006). Rh36 cells expressing wild-type p53 were incubated with either MI-63 or Nutlin-3, an MDM2 inhibitor, for 3 days. MTT assay showed a greater decrease in cell proliferation/viability by MI-63 as compared with a similar concentration of Nutlin-3 (Figure 8). The IC_50_ values obtained for MI-63 and Nutlin-3 were 0.58 *μ*M and 1.35 *μ*M, respectively.

### Quantitative combination effects between MI-63 and doxorubicin

**Figure 9:**
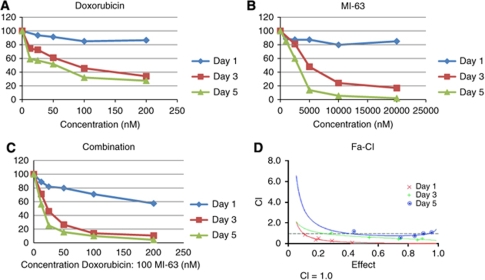
Inhibition of cellular proliferation in RH-36 cells by treatment with (**A**) doxorubicin, (**B**) MI-63, or (**C**) a combination of both, as measured by MTT analysis. (**D**) A CI-effect plot: CI values calculated from proliferation experiments were plotted against the effects (*fa*) of five different concentrations of the drugs.

Incubation of RH-36 cells with either doxorubicin or MI-63 at concentrations ranging from 12.5 to 200 nM or from 1250 to 40000 nM, respectively, led to a dose-dependent decrease of cellular viability as determined by the MTT assay (Figures 9A and B). IC_50_ values obtained at 1, 3, and 5 days after treatment were 8416, 82, and 34 nM for doxorubicin, and 576980, 5915, and 2407 nM for MI-63, respectively. MI-63 dosages of >20 *μ*M were saturated and no further decrease in viability was observed (data not shown); this may explain the exceptionally large IC_50_ values after 1 day of treatment. To determine the combinational effects of these two compounds, a constant molar ratio of 1 : 100, doxorubicin:MI-63, was used at all time points studied of the individual drugs (Figure 9C). A representative CI-effect plot for each time point is presented (Figure 9D). All concentrations at each time point tested have a CI value less than 1, indicating a synergism between doxorubicin and MI-63. At day 5, CI values approach 1, possibly indicating an additive effect; however, it must be noted that there was a nearly complete cell kill by day 5 in many of the concentrations. Overall, these data demonstrate a synergistic effect between doxorubicin and MI-63 that could prove useful in therapy to both improve survival and decrease toxicity.

## Discussion

Inhibition of p53 by MDM2 is known to have a role in tumour cell proliferation and in increasing oncogenesis. Recent studies have shown that targeted therapy directed at the p53–MDM2 interaction may have a therapeutic benefit (Ding *et al*, 2006; Tovar *et al*, 2006). In this experiment, we demonstrate that a novel small-molecule inhibitor, MI-63, has the ability to reactivate the wild-type p53 function in paediatric RMS. MI-63 targets the p53–MDM2 interaction, blocking the inhibition of the tumour suppressor and protein ubiquitination (Ding *et al*, 2006). After treatment, reactivation of wild-type p53 allows for downstream cell-cycle arrest and initiation of apoptosis in both ARMS and ERMS cell lines. Testing indicates that MI-63 has a minimal apoptotic effect on normal human tissue *in vitro*.

p53 is a tumour suppressor gene that regulates cell cycle, DNA repair, and apoptosis. Although specific mutations of the p53 protein have been described in approximately 50% of human cancers, a disruption of wild-type p53 transcriptional activity can independently contribute to tumourgenesis in other cancer types (Chene, 2004; Haupt and Haupt, 2004; Tovar *et al*, 2006). MDM2 is a regulator of p53 transcription activity. A direct binding to the p53 transcription activation domain inhibits G_1_ cell-cycle arrest and apoptosis signalling; however, with cellular stress, p53 activity is enhanced and appropriate signalling occurs (Chen *et al*, 1996). Increased activity of MDM2 contributes to defective, wild-type p53 regulation and can interrupt normal tumour suppressor activity, leading to malignant proliferation (Chen *et al*, 1996; Keleti *et al*, 1996).

There have been several reports of MDM2 amplification and/or overexpression in human cancers (Keleti *et al*, 1996; Momand *et al*, 1998; Klein and Vassilev, 2004; Takahashi *et al*, 2004; Levav-Cohen *et al*, 2005; Tovar *et al*, 2006). Momand *et al* examined nearly 4000 tumour samples and reported a 7% frequency of MDM2 amplification, with the highest observed in soft-tissue sarcomas (20%). Evaluation of RMS specifically suggests that an increased MDM2 activity is present in a sub-population of both human tissue samples and *in vitro* cell lines contributing to wild-type p53 inactivity (Keleti *et al*, 1996; Taylor *et al*, 2000; Takahashi *et al*, 2004). There seems to be no relationship between MDM2 amplification and p53 mutation, suggesting that MDM2 provides an alternative mechanism for inhibiting wild-type p53 (Keleti *et al*, 1996). Further, Taylor *et al* demonstrated the presence of wild-type p53 in 19 of 20 ERMS and ARMS tissue samples obtained either at the time of diagnosis or after chemotherapy. These findings draw attention to the p53–MDM2 interaction in RMS, suggesting that blocking MDM2 will reactivate wild-type p53.

The novel small-molecule inhibitor, MI-63, shows potential as an MDM2 antagonist. The potent, non-peptide inhibitor of the p53–MDM2 interaction is designed to mimic previously described hydrophobic residues (Phe19, Trp23, and Leu26), and a newly identified fourth residue (Leu22) in p53 that interacts with the hydrophobic cleft on MDM2 (Ding *et al*, 2006). With this novel design, MI-63 shows an increased binding (K_*i*_ 3 nM) to MDM2, and when compared with previously described non-peptide inhibitors (i.e., Nutlin-3), MI-63 is approximately 12 times more potent (Ding *et al*, 2006). Ding *et al* described a specific binding to MDM2, an increase in p53 levels, and the increase of downstream target p21^WAF1^ in adult prostate cancer cells (LNCAP) after treatment. The effect of MI-63 has also been observed in non-Hodgkin's lymphoma cell lines, in which similar results have been reported (Jones *et al*, 2008). Our experiment is the first to specifically analyse MI-63 and paediatric RMS (ERMS and ARMS) with wild-type p53.

The results demonstrate the effectiveness of MI-63 against RMS cell lines with wild-type p53. As hypothesised, both ERMS and ARMS cells (RH36 and RH18, respectively) had decreased cell viability after treatment. A previous evaluation of MI-63 demonstrated a reactivation of p53, but only described downstream increases in p21^WAF1^. We looked at both cell growth arrest and downstream indicators of apoptosis in ERMS and ARMS. Expectedly, p53 and p21^WAF1^ were elevated on western blot analysis. Pro-apoptotic markers, Bax, cleaved PARP, and cleaved caspase-3, all increased after treatment with MI-63 and may indicate a more comprehensive reactivation of p53. Notably, human cell lines treated similarly had minimal increases in p53 and p21^WAF1^ at higher doses of MI63; however, there was no evidence of decreased cell viability or apoptosis. Blocking MDM2 in normal cells understandably leads to a reflexive increase in p53, but importantly, this mechanism did not induce death. *In vivo* studies and phase I trials will better describe the short- and long-term effects of MI-63.

When treating RMS cells with MI-63 in combination with a known chemotherapeutic agent, doxorubicin, synergism was confirmed. Doxorubicin binds and intercalates DNA, inhibiting macromolecular synthesis by blocking the action of DNA topoismerase II. In addition, an inhibition of topoisomerase II prevents replication. As doxorubicin may act in a p53-independent manner, we hypothesised that a combination treatment with MI-63 would potentiate each drug's anti-proliferative effects (Taylor *et al*, 2000). The data agree with this supposition, most noted at early time points. Specifically, 20 nM of doxorubicin, in combination with 2000 nM MI-63 (day 1), showed a 49% increase in the fraction of cells affected by treatment when compared with the expected additive effect. *In vivo* studies, as well as clinical trials, are necessary to fully evaluate combinatorial effects and clinical usage.

In summary, we have shown that the novel small-molecule inhibitor, MI-63, successfully blocks MDM2 and reactivates p53 and downstream cell signalling when tested *in vitro*. Further, antitumour effects occur with minimal toxicity to normal cells and show synergism when combined with a known chemotherapeutic agent, doxorubicin. *In vivo* studies on the therapeutic potential of MI-63 are needed. Moving forward, as both RMS and other solid tumours (i.e., neuroblastoma) have wild-type p53 protein, clinical trials may focus on blocking p53–MDM2 interaction in solid tumours as a whole.
